# Subcutaneous application of hyperimmune serum against *Histophilus somni* recombinant proteins affects serum antibody reactivity in beef calves

**DOI:** 10.1186/s12917-024-03895-2

**Published:** 2024-02-10

**Authors:** Joanna Bajzert, Paulina Jawor, Rafał Baran, Tadeusz Stefaniak

**Affiliations:** Department of Immunology, Pathophysiology and Veterinary Preventive Medicine, Wroclaw University of Environmental and Life Sciences, C.K. Norwida 31 Str, Wrocław, 50-375 Poland

**Keywords:** OMP40, Hsp60, Immunity, Health protection

## Abstract

**Background:**

Respiratory tract diseases cause significant economic loss in beef cattle. This study aimed to determine whether the application of hyperimmune serum (HS) containing antibodies against selected antigens of Gram-negative bacteria would improve the health and growth of different breeds of beef calves kept on three farms. Two recombinant protein antigens (*Histophilus somni* rHsp60 and rOMP40) were used to immunize four cows to produce HS. Eighty seven beef calves (Charolaise *n* = 36, Limousine *n* = 34, and crossbreed *n* = 17) were included into study. One hundred milliliters of serum were administered subcutaneously to 43 beef calves (Charolaise *n* = 18, Limousine *n* = 17, and crossbreed *n* = 8) twice, between 1 and 5 and 21–28 days of life. Calves were examined three times, and blood samples were taken to evaluate immunoglobulin M, G_1_, and G2, fibrinogen, serum amyloid A, and haptoglobin concentrations and reactivity of these Ig classes of antibodies against *H. somni* rHsp60 and rOMP40. Average daily weight gain during the first month and until weaning was calculated.

**Results:**

HS showed higher (*p* ≤ 0.05) reactivity in calf sera against *H. somni* rHsp60 and OMP40 in IgG_1_ and IgG_2_. In experimental calves, compared to control calves, the reactivity of IgG_1_ against rOMP40 in the second sampling was higher in Limousine calves (*p* ≤ 0.001) and in the other two herds (*p* ≤ 0.05). Serum IgG_2_ antibody activity against *H. somni* rHsp60 in the second sampling was higher in experimental calves than in control calves in charolaise (*p* ≤ 0.05) and limousine (*p* ≤ 0.001) herds. The reactivity of IgG_2_ against rOMP40 in the second sampling of experimental calves was higher in herds with Charolaise and Limousine calves (*p* ≤ 0.001) and in crossbred calves (*p* ≤ 0.05). In the third sampling, serum IgG_1_ antibody reactivity against rOMP40 in Limousine calves was higher (*p* ≤ 0.05) in the experimental group. Among the other evaluated parameters, only SAA in the second sampling in the herd with Charolaise calves and heart rate in the herd with Limousine calves were significantly higher in the control calves (*p* ≤ 0.05).

**Conclusion:**

The application of HS to calves in all herds had an impact on specific reactivity in IgG_1_ and IgG_2_ classes against *H. somni* rOMP40 and rHsp60, antigens which were used for serum production.

**Supplementary Information:**

The online version contains supplementary material available at 10.1186/s12917-024-03895-2.

## Introduction

During the rearing period, respiratory tract diseases are the most expensive illness in beef cattle and are a major health problem in feedlot cattle [1]. Maintaining health is crucial for improving the growth rate and effectiveness of beef production [2]. Metaphylactic antimicrobial programs are used to prevent and treat bovine respiratory disease (BRD); however, this strategy fails with respect to the prudent use of antimicrobial treatments, promoting the selection of resistance gene determinants and antimicrobial-resistant bacteria [3]. Commercial polyvalent immune serum against *Escherichia coli, Salmonella* Dublin and *Salmonella* Typhimurium given IV (intravenously) to colostrum-deprived calves protected them against death after oral challenge with *E. coli* O78:K80(B) [4]. The administration of hyperimmune plasma, especially in cases of passive transfer failure, is advisable for the prophylaxis and treatment of neonatal calf diarrhea, which reduces the use of antibiotics [5]. Commercially available bovine hyperimmune serum (HS) administered to calves at the time of arrival to a feedlot in doses 3–6 times lower than the recommended label did not affect the incidence and severity of BRD or the number of days of treatment [6]. However, serum produced against *Histophilus somni* by cow immunization reduced the incidence of BRD in calves and reduced mortality [7].

*Histophilus somni* is among the most commonly implicated bacterial pathogens identified using culture methods in BRD cases [8]. Sera obtained from cattle, swine, dogs, horses, and poultry vaccinated with whole *H. somni* cells revealed a strong immune response against selected *H. somni* antigens, including major outer membrane proteins [9]. The outer membrane of *H. somni* is composed of a wide range of proteins, the most abundant of which is the major outer membrane protein (MOMP) called OMP 40 kDa (OMP40) [10]. The cross-reactivity of rabbit antiserum with whole-cell antigens of different *H. somni* strains revealed a reaction with proteins with a molecular mass of approximately 40 kDa [11]. Heat shock protein (HSP) 60 is a highly conserved protein that is widely distributed in nature and found in all prokaryotic and eukaryotic cells. Particularly in bacteria, the expression of Hsp60 on the surface occurs constitutively and increases remarkably during host infection [12]. Immunization of mice with one rHsp60 derived from four common pathogenic bacteria, *E. coli, H. somni, Pasteurella multocida*, and *Salmonella* Enteritidis, led to the production of antibodies that reacted with their homologous and heterologous antigens [13]. Mouse anti-rHsp60 *H. somni* antibodies have been shown to inhibit in vitro biofilm production by these bacteria [14]. Therefore, two protein antigens (*H. somni* rHsp60 and rOMP40) were used to immunize cows to produce HS. This study aimed to determine whether the application of HS improves the health status and increases the growth of different breeds of beef calves on three selected farms.

## Results

### Formulation protein analysis

**Fig. 1:**
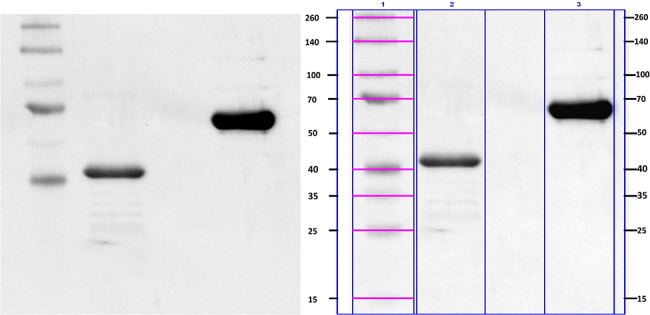
Western blot analysis of recombinant proteins *H.somni* OMP40 and *H.somni* Hsp60 used for cows immunization. First lane- Spectra™ Multicolor Broad Range Protein Ladder, Fermentas (3 µl/lane), second lane rOMP40 (1.5 µg/lane), third lane rHsp60 (1.5 µg/lane). Left image: raw; right image with masses of individual bands marked

Western blot analysis showed high purity of the obtained proteins. The molecular weights of the examined proteins were calculated as 41.55 kDa for rOMP40 and 62.03 kDa for rHsp60 (Fig. 1).

### Cows’ hyperimmunization

Two weeks after the first immunization (S2), significantly higher reactivity against rHsp60 and rOMP40 was detected in IgG1 and IgG2 than in S1. This difference was observed until the end of immunization. No significant increase in the reactivity of the IgM class antibody was observed (Table 1).

**Table 1:** Median (mean ± SD) cows’ serum reactivity with recombinant proteins in ELISA during hyperimmunization

Antigen	Ig class							
		S1 *n* = 8	S2 *n* = 8	S3 *n* = 8	S4 *n* = 8	S5 *n* = 8	S6 *n* = 8	Final serum *n* = 6
**rHsp60**	**IgG1**	0.15 (0.15 ± 0.0)	0.26* (0.29 ± 0.1)	1.02* (1.00 ± 0.3)	1.25* (1.31 ± 0.3)	1.24* (1.23 ± 0.1)	1.24* (1.29 ± 0.3)	0.93* (1.13 ± 0.3)
	**IgG2**	0.14 (0.14 ± 0.0)	0.33* (0.30 ± 0.1)	1.05* (1.09 ± 0.5)	1.50* (1.46 ± 0.5)	1.68* (1.61 ± 0.5)	1.62* (1.65 ± 0.2)	1.96* (1.71 ± 0.6)
	**IgM**	0.82 (0.79 ± 0.2)	0.82 (0.81 ± 0.2)	0.82 (0.85 ± 0.1)	0.81 (0.81 ± 0.1)	0.85 (0.87 ± 0.1)	0.87 (0.89 ± 0.2)	0.68 (0.69 ± 0.1)
**rOMP40**	**IgG1**	0.15 (0.15 ± 0.0)	0.32* (0.44 ± 0.4)	0.99* (1.03 ± 0.2)	0.90* (1.02 ± 0.4)	1.20* (1.09 ± 0.3)	1.15* (1.14 ± 0.3)	1.16* (1.13 ± 0.2)
	**IgG2**	0.16 (0.19 ± 0.1)	0.29* (0.77 ± 1.0)	1.27* (1.28 ± 0.7)	1.29* (1.32 ± 0.7)	1.31* (1.31 ± 0.5)	1.36* (1.35 ± 0.7)	1.47* (1.37 ± 0.4)
	**IgM**	0.53 (0.52 ± 0.2)	0.65 (0.72 ± 0.2)	0.79 (0.91 ± 0.4)	0.77 (0.92 ± 0.4)	1.11 (0.98 ± 0.3)	0.95 (0.94 ± 0.4)	0.76 (0.92 ± 0.3)

Legend: n- number of optical density results analyzed, *significantly different from S1 at *p* ≤ 0.05. rHsp60 - *H. somni* recombinant heat shock protein, rOMP40- *H. somni* recombinant outer membrane protein; S1-S5subsequent sampling before immunizations S3- three weeks after last immunization

### Laboratory results from calves

No significant differences in the reactivity of the IgM class against rHsp60 and rOMP40 and IgG_1_ against rHSP60 in subsequent samplings were detected in the experimental compared to the control calves (Table 2).

**Table 2:** Calves sera reactivity in the IgM and IgG_1_ class with evaluated proteins in ELISA

		Herd	CH		LM		MIX	
Protein	Ig class	Sampling	C *n* = 18	E *n* = 18	C *n* = 17	E *n* = 17	C *n* = 9	E *n* = 8
**rHsp60**	**IgM**	**1**	1.25 (0.97–1.60)	1.38 (1.11–1.67)	1.04 (0.67–1.31)	0.77 (0.49–1.23)	0.77 (0.53–0.90)	0.87 (0.47–1.07)
		**2**	0.44 (0.34–0.51)	0.47 (0.41–0.53)	0.27 (0.23–0.31)	0.26 (0.21–0.30)	0.24 (0.08–0.29)	0.29 (0.09–0.34)
		**3**	1.27 (0.97–1.87)	1.30 (1.10–1.52)	1.61 (1.42–2.01)	1.81 (1.52–2.12)	1.94 (1.19–2.61)	2.55 (2.00–3.00)
**rOMP40**		**1**	0.70 (0.57–1.03)	0.90 (0.66–1.36)	0.58 (0.40–0.81)	0.49 (0.41–0.55)	0.42 (0.37–0.47)	0.53 (0.33–0.59)
		**2**	0.34 (0.30–0.42)	0.38 (0.31–0.53)	0.28 (0.23–0.31)	0.30 (0.24–0.33)	0.25 (0.21–0.26)	0.21 (0.18–0.31)
		**3**	0.87 (0.70–1.05)	0.87 (0.71–1.17)	1.02 (0.91–1.13)	1.06 (0.93–1.25)	1.64 (0.94–2.24)	1.47 (0.91–1.97)
**rHsp60**	**IgG**_**1**_	**1**	1.24 (1.06–1.58)	1.37 (0.99–1.78)	0.93 (0.62–1.52)	0.80 (0.59–1.14)	0.83 (0.59–0.92)	0.66 (0.43–1.11)
		**2**	0.87 (0.62–1.18)	1.04 (0.93–1.42)	0.69 (0.52–0.88)	0.82 (0.61–1.06)	0.46 (0.33–1.08)	0.67 (0.43–0.99)
		**3**	0.84 (0.57–1.29)	0.71 (0.43–1.35)	1.42 (1.00-1.70)	1.04 (0.81–1.48)	0.69 (0.56–0.75)	0.48 (0.34–0.64)

Legend: Median (Q1-Q3) optical density. rHsp60, rOMP40- *H. somni* recombinant proteins. Herds (dominant cows breed) CH- Charolaise, LM-limousine, MIX crossbreed beef and dairy breed. C- control, E- experimental

**Fig. 2:**
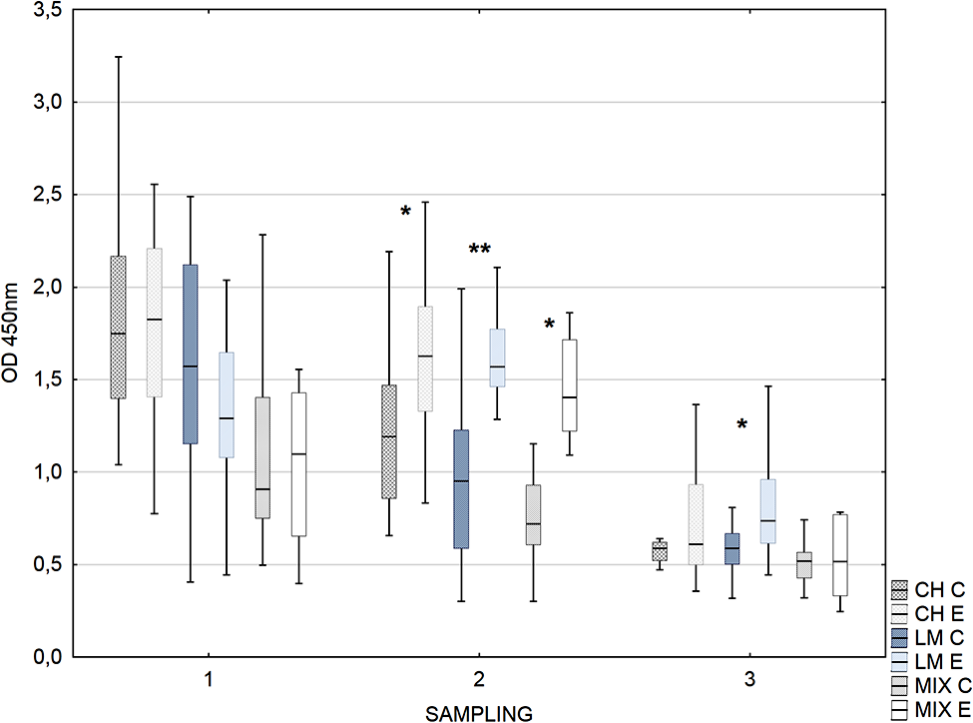
Box-plot graph of calves sera IgG_1_ antibody reactivity against *H. somni* rOMP40. The median line across the box, lower and upper boxes indicates the Q1-Q3, whisker-nonoutlier range. Groups with * differ at *p* ≤ 0.05. Groups with ** differ at *p* ≤ 0.001. Herds (dominant cows breed) CH- Charolaise, LM-limousine, MIX crossbreed beef and dairy breed. C- control, E- experimental

The reactivity of IgG_1_ against rOMP40 in the second sampling was significantly higher in experimental calves than in control calves in all examined herds (Fig. 2). At S3, serum IgG_1_ reactivity in experimental calves was significantly higher than in control calves in the LM herd.

**Fig. 3:**
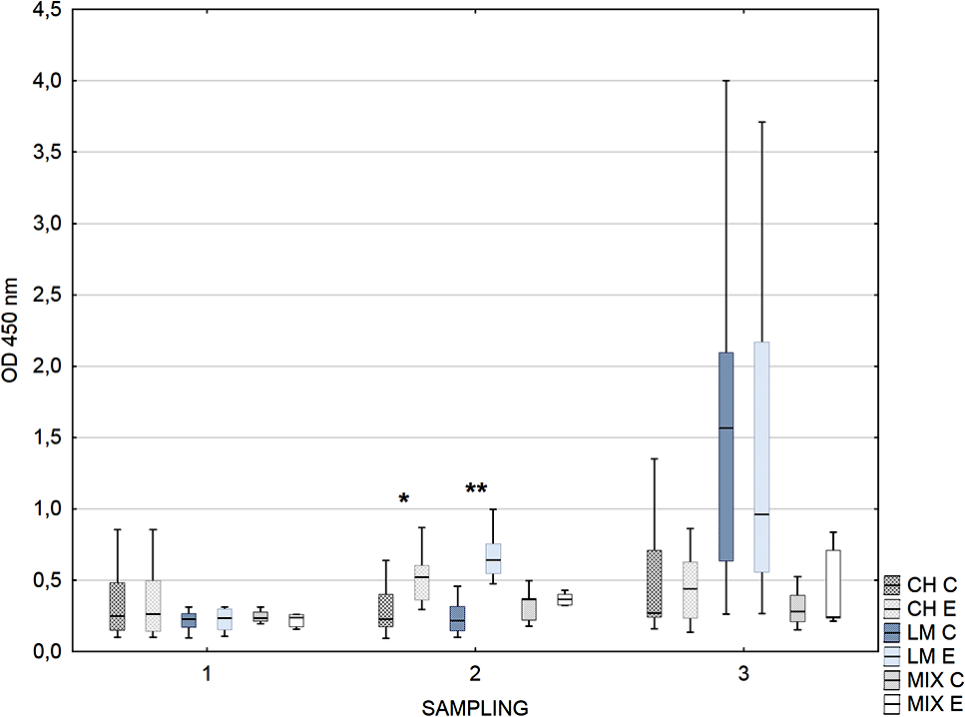
Box-plot graph of calves sera IgG_2_ antibody reactivity against *H. somni* rHsp60. Legend: Median line across the box, lower and upper boxes indicating the Q1-Q3, whisker- nonoutlier range. Groups with * differ at *p* ≤ 0.05. Groups with ** differ at *p* ≤ 0.001. Herds (dominant cows breed) CH- Charolaise, LM-limousine, MIX crossbreed beef and dairy breed. C- control, E- experimental

The reactivity of the IgG_2_ antibody against rHsp60 in the second sampling was significantly higher in experimental calves than in control calves in herds CH and LM (Fig. 3).

**Fig. 4:**
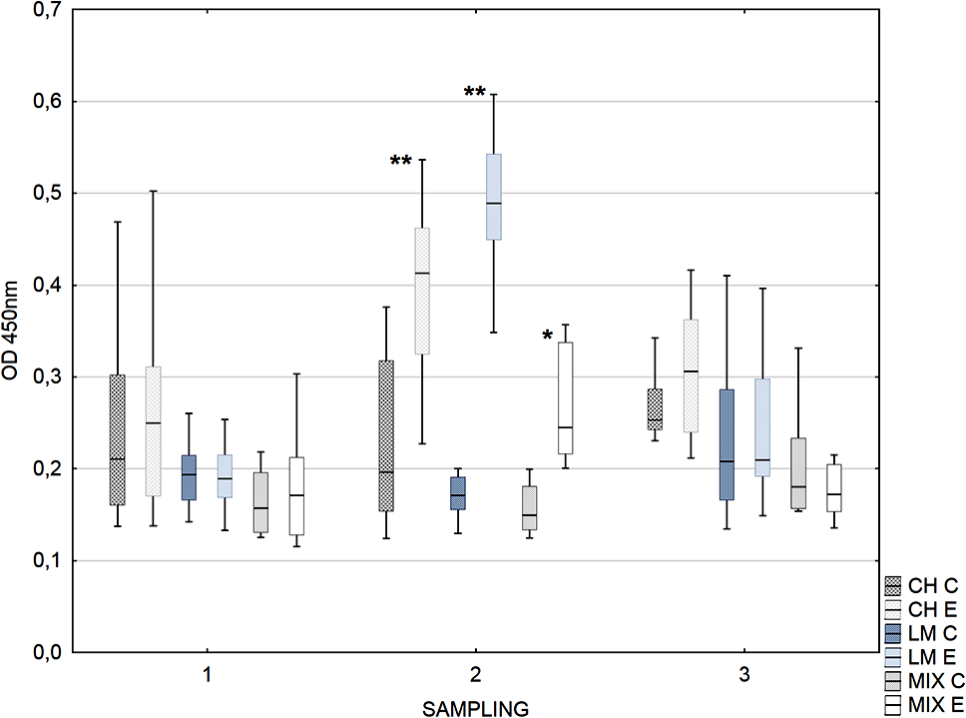
Box-plot graph of calves sera IgG_2_ antibody reactivity against *H. somni* rOMP40. Legend: Median line across the box, lower and upper boxes indicating the Q1-Q3, whisker- nonoutlier range. Groups with * differ at *p* ≤ 0.05. Groups with ** differ at *p* ≤ 0.001. Herds (dominant cows breed) CH- Charolaise, LM-limousine, MIX crossbreed beef and dairy breed. C- control, E- experimental

The reactivity of the IgG_2_ antibody against rOMP40 in the second sampling was significantly higher in experimental calves than in control calves in all examined herds (Fig. 4).

**Table 3:** The concentration of serum immunoglobulins, acute phase proteins, rectal temperature, respiratory and heart rates

		CH		LM		MIX	
Parameter	Sampling	C *n* = 18	E *n* = 18	C *n* = 17	E *n* = 17	C *n* = 9	E *n* = 8
**IgG**_**1**_ **g/L**	**S1**	22.0 (22.3 ± 6.9)	26.4 (26.3 ± 9.9)	17.2 (15.2 ± 8.2)	11.2 (15.5 ± 9.8)	11.4 (13.4 ± 5.8)	12.2 (13.4 ± 8.1)
	**S2**	18.1 (17.4 ± 6.7)	16.2 (16.7 ± 7.1)	10.4 (11.4 ± 5.7)	10.6 (10.7 ± 4.7)	9.8 (10.5 ± 3.2)	9.1 (9.3 ± 4.0)
	**S3**	11.0 (11.2 ± 2.7)	10.8 (11.3 ± 2.2)	8.4 (8.5 ± 1.2)	8.2 (8.3 ± 1.6)	12.0 (13.0 ± 4.8)	8.4 (11.4 ± 4.8)
**IgG**_**2**_ **g/L**	**S1**	1.1 (1.4 ± 0.9)	1.4 (1.5 ± 0.9)	1.1 (1.3 ± 0.9)	0.9 (1.0 ± 0.6)	0.6 (0.8 ± 0.6)	0.6 (0.6 ± 0.4)
	**S2**	0.9 (1.0 ± 0.5)	1.0 (1.1 ± 0.4)	0.6 (0.7 ± 0.4)	0.8 (0.8 ± 0.4)	0.5 (0.7 ± 0.5)	0.6 (0.6 ± 0.2)
	**S3**	3.0 (3.5 ± 2.1)	2.6 (3.1 ± 2.0)	3.0 (3.5 ± 2.3)	3.0 (3.2 ± 1.5)	2.9 (3.2 ± 1.0)	3.4 (3.3 ± 0.9)
**IgM** **g/L**	**S1**	2.1 (2.1 ± 0.6)	2.2 (2.4 ± 1.3)	1.5 (1.5 ± 1.0)	1.1 (1.1 ± 0.8)	0.8 (1.0 ± 0.7)	0.9 (1.0 ± 0.7)
	**S2**	0.5 (0.5 ± 0.3)	0.5 (0.6 ± 0.3)	0.3 (0.4 ± 0.3)	0.3 (0.3 ± 0.1)	0.4 (0.4 ± 0.3)	0.3 (0.3 ± 0.1)
	**S3**	2.2 (2.0 ± 0.6)	1.8 (1.8 ± 0.7)	1.4 (1.5 ± 0.5)	1.4 (1.5 ± 0.6)	2.1 (2.2 ± 0.9)	2.2 (2.1 ± 0.7)
**Fb** **g/L**	**S1**	4.8 (4.5 ± 1.4)	4.6 (4.7 ± 1.3)	5.3 (5.2 ± 0.8)	5.8 (5.6 ± 1.4)	5.7 (5.5 ± 1.1)	5.5 (5.6 ± 1.3)
	**S2**	4.5 (4.4 ± 1.6)	4.3 (4.3 ± 1.1)	4.3 (4.2 ± 1.1)	4.7 (4.6 ± 1.2)	5.3 (5.5 ± 1.2)	5.6 (5.8 ± 1.5)
	**S3**	4.9 (4.7 ± 0.8)	4.7 (4.5 ± 1.0)	4.8 (5.1 ± 1.1)	4.7 (4.7 ± 0.7)	4.9 (5.0 ± 1.2)	5.0 (5.2 ± 1.0)
**SAA** **mg/L**	**S1**	58.8 (61.0 ± 18.6)	60.8 (68.8 ± 26.5)	85.2 (93.7 ± 33.6)	69.1 (76.4 ± 24.3)	58.5 (74.0 ± 35.0)	92.8 (87.7 ± 38.6)
	**S2**	23.4* (29.6 ± 18.0)	15.2* (18.7 ± 12.8)	22.8 (28.6 ± 19.3)	32.7 (29.8 ± 16.4)	43.9 (35.2 ± 16.6)	36.3 (35.8 ± 16.9)
	**S3**	4.6 (12.0 ± 18.4)	3.2 (10.3 ± 20.5)	10.6 (19.3 ± 22.5)	6.3 (15.8 ± 22.7)	0.9 (2.9 ± 3.9)	3.7 (8.7 ± 11.7)
**Hp** **mg/L**	**S1**	5.3 (5.3 ± 1.6)	5.0 (7.7 ± 10.8)	4.3 (7.2 ± 8.4)	6.4 (11.9 ± 20.2)	4.7 (11.4 ± 19.2)	12.7 (22.7 ± 25.0)
	**S2**	5.2 (9.3 ± 15.0)	5.1 (4.3 ± 2.2)	5.5 (7.9 ± 15.1)	5.1 (9.0 ± 13.6)	4.5 (6.4 ± 6.3)	5.9 (53.3 ± 123.3)
	**S3**	5.2 (5.8 ± 1.4)	5.8 (6.2 ± 1.7)	5.4 (9.0 ± 14.0)	5.4 (19.0 ± 54.9)	6.0 (6.7 ± 2.0)	5.0 (5.4 ± 0.9)
**Rectal temperature** ^**o**^ **C**	**S1**	38.9 (39.0 ± 0.4)	39.0 (39.0 ± 0.3)	39.3 (39.3 ± 0.3)	39.1 (39.1 ± 0.4)	39.2 (39.1 ± 0.3)	39.0 (39.0 ± 0.3)
	**S2**	39.1 (39.1 ± 0.4)	39.0 (39.0 ± 0.2)	39.7 (39.5 ± 0.3)	39.5 (39.5 ± 0.3)	39.3 (39.2 ± 0.4)	39.2 (39.4 ± 0.8)
**Resp. rate/min**	**S1**	54.0 (57.3 ± 17.3)	52.0 (50.9 ± 11.7)	60.0 (58.1 ± 12.7)	48.0 (54.6 ± 23.1)	48.0 (49.8 ± 13.3)	46.0 (47.0 ± 10.8)
	**S2**	50.0 (61.8 ± 33.6)	52.0 (50.9 ± 9.5)	68.0 (70.1 ± 18.3)	64.0 (67.1 ± 17.1)	52.0 (56.4 ± 15.8)	48.0 (54.0 ± 17.4)
**HR/min**	**S1**	146.0 (146.4 ± 25.5)	144.0 (141.3 ± 29.1)	180.0* (182.6 ± 31.3)	156.0* (157.3 ± 22.9)	177.0 (171.3 ± 29.7)	156.0 (158.5 ± 24.5)
	**S2**	140.0 (139.1 ± 18.9)	146.0 (146.7 ± 14.7)	180.0 (183.3 ± 20.2)	188.0 (183.9 ± 22.4)	170.0 (167.6 ± 25.4)	166.0 (171.5 ± 21.4)

Legend: Values are median (mean ± SD). Groups with * differ at *p* ≤ 0.05, SAA- serum amyloid A, Hp- haptoglobin, Fb- fibrinogen, HR- heart rates. Herds (dominant cows breed) CH- Charolaise, LM-limousine, MIX crossbreed beef and dairy breed. C- control, E- experimental

Inadequate passive transfer, defined as < 24 g IgG/L [15], calculated by summing the concentrations of IgG_1_ and IgG_2_ classes, was present in 41.7%, 85.3%, and 94.1% of the CH, LM, and MIX herds, respectively.

No differences were observed between the experimental and control groups for IgG_1_, IgM, Hp concentration, rectal temperature, or respiratory rate in subsequent samplings (Table 3).

Serum IgG_2_ concentration was not markedly different between experimental and control calves in herds in subsequent samplings (Table 3). However, there was a significant interaction between herds and sampling time (*p* ≤ 0.01). Simple main effect analysis demonstrated that time S1 and S2 were significant, but not S3. Post hoc Tukey’s HSD for unequal N showed that in S1, the serum IgG_2_ concentration was significantly higher in Charolaise calves than in the MIX herd (1.4 ± 0.9 vs. 0.7 ± 0.5 g/L *p* < 0.01). In S2, Charolaise calves still had significantly higher serum IgG_2_ concentrations (1.0 ± 0.5 g/L) than Limousine and MIX calves (0.8 ± 0.4 and 0.7 ± 0.4 g/L, respectively, *p* < 0.05).

The Fb concentration was not significantly different between the experimental and control calves in subsequent samplings (Table 3). The Fb concentration was influenced by herd and sampling time (*p* < 0.01). Post hoc Tukey’s HSD for unequal N showed that Charolaise calves had significantly lower Fb concentrations than mixed calves (4.5 ± 1.2 vs. 5.4 ± 1.2 g/L, *p* < 0.01). The Fb concentration in the first sampling was significantly higher than that in the second sampling (5.1 ± 1.3 vs. 4.6 ± 1.3 g/L, *p* < 0.001).

SAA concentration was significantly higher in control calves compared to the experimental group in the second sampling only in the Charolaise herd (*p* < 0.05) (Table 3). Heart rate was significantly higher in the control than in the experimental Limousine calves in the first sampling (*p* < 0.05) (Table 3).

### Average daily gain

**Table 4:** Average daily gain kg/d ± SD in the first month and up to weaning

Herd		CH		LM		MIX	
Time	SEX	C	E	C	E	C	E
First month	Heifer	1.23 ± 0.16	1.17 ± 0.22	1.02 ± 0.29	1.12 ± 0.20	0.97 ± 0.38	1.03 ± 0.18
	Bull	1.25 ± 0.26	1.15 ± 0.47	1.11 ± 0.26	0.87 ± 0.24	1.03 ± 0.43	1.30 ± 0.12
> 1 month up to weaning	Heifer	1.31 ± 0.07	1.33 ± 0.15	1.14 ± 0.27	1.15 ± 0.13	0.98 ± 0.17	0.94 ± 0.08
	Bull	1.34 ± 0.26	1.49 ± 0.09	1.31 ± 0.15	1.27 ± 0.14	1.08 ± 0.04	1.14 ± 0.06

Legend: CH- Charolaise, LM-limousine, MIX crossbreed beef and dairy breed. C- control, E- experimental. First month weight difference between first weighting and S2 weighting

The application of serum did not influence ADG in the first month of life (Table 4). In the first month, there were no differences between herds and the sex of particular calves. There was no impact of serum application on ADG from the first month of age until weaning (Table 4), but ADG was influenced by herd type and sex. In the Charolaise herd, the ADG (1.36 ± 0.17 kg/d) was significantly higher (*p* < 0.01) than in the LM and MIX herds (1.21 ± 0.19; 1.00 ± 0.14, kg/d, respectively). The ADG of calves in the LM herd was significantly higher than that in the MIX herd (*p* < 0.01). In all herds, bull calves had significantly higher ADG than heifer calves (1.33 ± 0.19 vs. 1.17 ± 0.21, *p* < 0.001). The mean age at weaning ± SD was 157 ± 47 days in the CH herd, 186 ± 41 days in the L herd, and 231 ± 32 days in the MIX herd.

### Morbidity and mortality

In herd LM, three calves were treated due to bloat (C group), bloody diarrhea (C group), and omphalophlebitis (E group). Two calves died during the experimental period in herd LM. One was found dead on pasture (E group) and rumen bloat-previously treated (C group). There were no more treatment or death cases in the remaining herds.

## Discussion

In the following study, hyperimmune serum against two recombinant *H. somni* (rHsp60 and rOMP40) proteins was produced and administered subcutaneously to beef calves from three herds twice in the first month of life. *H. somni* Hsp60 is a heat shock protein that is widespread in nature and is an essential protein for growth and in the stationary phase of many bacteria [16]. Epitopes of the bacterial 60-kDa heat shock protein are present in various animal and human cells [17], which, according to some studies, may even trigger autoimmune reactions [18]. *H. somni* OMP40 is the major outer membrane protein [10]. During bacterial infection, some outer membrane proteins are recognized. It was shown that convalescent sera from calves with *Histophilus somni* (formerly *Haemophilus somnus)* pneumonia strongly reacted with outer membrane proteins (78 and 40 kDa) of *H. somni* in western blot [19]. Therefore, it may be expected that older cows, such as those used for immunization (≥ 5 years), which probably experienced different infections, will show serum IgM antibody reactivity against these two proteins. Jankowska et al. [20] demonstrated that cattle were able to respond very quickly (within three weeks) in IgG_1_ and IgG_2_ but not the IgM class to rHsp60 *H. somni* immunization. A similar observation was obtained in calves immunized with rOMP40 [21]. In this study, hyperimmunization with *H. somni* rHsp60 and rOMP40 resulted in significant immune responses in the IgG_1_ and IgG_2_ classes. This is in agreement with the findings of Chaiyotwittayakun et al. [22], where to induce increased titers and cross-reactivity to heterologous gram-negative bacteria, three or more repeated immunizations were necessary. Here, to produce a serum that possesses strong, stable antigen-specific recognition and cross-reactivity with other bacteria, we decided to vaccinate cows five times. Although a high response in both IgG subclasses was already present in S3 (two weeks after the second immunization), it should be remembered that the pattern of immunoglobulin response in isotypes and classes (Th1 and Th2 responses) in cattle depends on different factors, such as antigen dose, type and affinity for T-cell receptors and the cytokine environment during antigen priming, which can be influenced by the nature of the pathogen or antigen [23].

Increased reactivity of IgG_2_ and IgG_1_*H. somni* rOMP40 and rHsp60 antibodies at the end of the first month of life may be expected in calves receiving hyperimmune serum subcutaneously, as the half-life of IgG serum immunoglobulin was calculated to be 27 days [24], and hyperimmune serum showed significantly higher reactivity of specific antibodies. The significant difference between groups C and E in IgG_1_ serum antibody reactivity was also noted in S3 (before weaning) but only in the LM herd. This is surprising since the previously reported IgG_1_ half-life is shorter than that for IgG_2_ [25]. IgG_1_ is more efficient as an opsonin than IgG_2_, and both activate the bovine complement cascade [26, 27]. The IgG_2_ subclass is crucial in protection against pyogenic infections [28, 29]. Therefore, an increase in the reactivity of these subclasses may enhance the ability of calves to fight infection. In this study, differences in rHsp60 IgG_2_ antibody reactivity between the control and experimental groups were noted in S2 in the CH and LM but not in the MIX herd. This may be because the ELISA antibody reactivity with *H. somni* rOMP40 and rHsp60 in the cow donor from herd MIX (the oldest animal in the study) was almost 50% lower than that in other hyperimmunized cows (data not shown).

In each herd, control and experimental calves had similar concentrations of serum immunoglobulins. The numerically highest concentration of IgG_1_ and statistically higher IgG_2_ concentration were detected in herd CH, where calves were first fed from a bottle with colostrum milked from the dam. Beef calves with serum IgG concentrations < 24 g/L are considered to have inadequate transfer of passive immunity due to their increased risk of preweaning morbidity and mortality [15, 30]. In this study, no significant mortality was detected in the examined herds. Increased SAA, but not Hp or Fb concentrations in S2 in control calves in the CH herd, may suggest the presence of an inflammatory process, as SAA is more sensitive in detecting the inflammatory process than Hp [31]. However, the median SAA concentration was below the threshold for SAA in healthy calves, i.e., 25.6 mg/L [32]. ADG was not influenced by experimental design but by genetics [33] and sex [34], as well as possibly because calves in the CH herd were also fed concentrate in addition to pasture [35], in contrast to other herds.

## Conclusions

The application of hyperimmune serum to calves in the examined herds did not affect any measured parameters, except for an increase in specific reactivity in IgG_1_ and IgG2 classes against proteins that were used for serum production. This was observed mainly in the first month after serum administration. Calf morbidity and mortality were low in all herds. Further studies are necessary to evaluate the clinical effect of the described procedure in herds with a common incidence of such infections.

## Materials and methods

### Herds

The study was carried out in three herds (CH, LM, MIX). Animals were privately owned by farms. In two herds, calves of beef breeds and in one crossbreed (dairy and beef breed) were reared up to weaning. The dominant cows breed in the CH herd was > 50% Charolaise and mixed with other breeds (Simmental, Salers). The cows were inseminated with the semen of Charolaise bulls. In herd LM, the cows were 100% limousine breed and were inseminated with the semen of Limousine bulls. In the MIX herd, the dams were mixed with different proportions of dairy breeds (Holstein Friesian red factor and Polish Red and White) with the Limousine breed. All cows in this herd were inseminated with the semen of limousine bulls. The cows were not vaccinated against infectious diseases in any herd.

On all farms, the grazing season was from May to October, and in the remaining period of the year, animals were kept indoors in a loose housing system with straw bedding. During the indoor period, cows were fed with hay, grass silage, and corn silage (in LM, MIX herds) or hay, alfalfa silage, and whole crop silage from rye and oat (in CH herd). Only calves born from multiparous cows were included in the study (the number of pregnant heifers in all herds was low, 1–3 per herd). The calving season in all farms started at the end of January, and the last calf was born in June.

#### Procedures in the herd “CH”

Cows were separated from others as signs of approach parturition were recognized (relaxation of pelvic ligaments and swelling of the vulva). After the calving dam was kept either in an individual pen or with another cow for the next 2–3 days, the dam and offspring were reunited with the herd. Each calf within 2–3 h after birth was fed with hand-milked colostrum in the amount of 1-1.5 L. Calves were fed first with colostrum from the bottle with a nipple, and then the calf was left with the dam. In two cases (death of mother, no colostrum due to previously milked by another calf), frozen colostrum was used. After animals were reunited with the herd, the calves had access to a pen with a selection gate to which cows had no access. In this area, the calves had unlimited access to musli (Kälber Starter Musli, Blattin) and hay. During the grazing season, calves were additionally fed with a pellet for young beef cattle (18% total protein, 1.7% fat, 5.8% crude fibre, 7.75% crude ash).

#### Procedures in herd “LM”

The calves were born in group pens, where all cows were kept. The dam’s behavior was observed to detect if she was nursing. If the mother did not allow the calf to suck the udder, a cow was tied up for the time of nursing (one case). The herd did not use frozen colostrum. The calves had access to separate pens that the cows were not allowed (selection gate). In that pen, the calves had free access to the feed mixture for the calves. At the beginning of the calving season, pelleted prestarter was given (23.0% total protein, 3.5% fat, 7.0% crude fibre), and two months later, pellets were given to older calves (17% total protein, 1.9% fat, 4.86% crude fibre). During the grazing season, the calves were not additionally fed.

#### Procedures in herd “MIX”

As signs of approaching parturition were noted (relaxation of pelvic ligaments and swelling of the vulva), the dams were separated from the herd and kept either in individual pens or with another cow for the time of calving and the next 2–3 days, after which the dam and calf were reunited with the herd. The first nursing of the calf was supervised; if it was unable to suck alone, it was assisted. The herd did not use frozen colostrum. The calves did not have a separate pen, but they were able to reach the feeding bunk, where straw bedding was placed on one end. In that area, calves had access to prestarter and later pellet. At the beginning of the calving season, pelleted prestarter was given (23.0% total protein, 3.5% fat, 7.0% crude fibre), and two months later, pellets were given to older calves (17% total protein, 1.9% fat, 4.86% crude fibre). During the grazing season, the calves were not additionally fed.

In none of the herds was the quality of colostrum measured.

### Hyperimmune serum production

One cow from the LM (5 years old) and MIX farms (12 years old) and two cows from the CH herd (8 and 10 years old) were selected for serum production. The number of animals was selected due to the predictable number of calves that will be born in the evaluated calving season.

Cows were immunized subcutaneously with a formulation containing 10 µg of *H. somni* rHsp60 protein + 20 µg of *H. somni* rOMP40 protein + Emulsigen adjuvant 20% (v/v) and saline (given up to a total volume of one dose of 1000 µl). Five immunizations were performed at 12-14-day intervals. Each time, the animals received 1 ml of vaccine SC in the lateral neck, alternating right and left side for each vaccination. Samples of blood from the tail vein were taken to obtain serum and plasma before each immunization to control the effect of the process (samples S1-S4). Immediately before the fifth immunization and three weeks later, 5 L of blood was taken from the jugular vein of each cow (samples S5-S6). Blood was collected in 15 L plastic disinfected buckets. Blood was immediately manually mixed for 15 min with stainless steel tongs to eliminate fibrinogen. In the laboratory, the blood was centrifuged in RT for 20 min at 1100 x g to separate the blood cells from the serum, which was then collected. The obtained serum within 5 h was phenol-preserved to achieve a final concentration of 0.5% and poured into 250 ml glass bottles kept in 4 ^*o*^*C*. Serum was produced separately for each farm.

### Treatment groups

Newborn calves were alternately allocated to the experimental (hyperimmune serum was given) and control groups (without serum treatment). If the sex ratio in each group was uneven, the next calf of the appropriate sex was added to the group in which the number of calves of this sex was lower.

Each calf was weighed within 24 h of birth. The duration of the study began with sampling (S1) and ended when the calves were weaned (sampling S3). The calves were enrolled in the study between 24 and 114 h after birth (first sampling – S1). At S1, clinical examination of each calf was performed. It included breathing rate measured from distance, internal rectal temperature, heart rate (with a stethoscope), auscultation of the lungs (presence of abnormal sounds), examination of the umbilical cord (thickness and painfulness), presence (yes/no), and characteristics of nasal and eye discharge (serous, mucous, purulent). After the calf examination, blood from the jugular vein was collected into 2 ml EDTA tubes (Improvacuter, China) and 9 ml tubes to obtain serum (Vacuette, Austria). Hyperimmune serum in a total volume of 100 ml was given SC to experimental calves (50 ml to each side of the chest). The second blood sampling (S2) was between 21 and 28 days of calf age. During the S2 visit weighing, clinical examination (as in S1) and blood collection were performed, and another dose of the serum (100 ml) was given to the experimental calves (50 ml of each side of the chest subcutaneously). During the third blood sampling, before weaning (S3), only weighing and blood sampling were performed. During each visit, noticed cases of calf diseases were recorded. The average daily gain (ADG, kg/day) was calculated based on the difference in body weight between each weights (first within 24 h of life, second at S2 and third at S3 visit) divided by the number of days elapsed between the weighing dates. Weighing was performed using an electronic scale. In the CH herd, there were 18 calves (8 bulls) in the experimental group and 18 calves in the control group (8 bulls). In the LM herd, there were 17 calves (7 bulls) in the experimental group and 17 calves in the control group (8 bulls). In the MIX herd, there were 8 calves (2 bulls) in the experimental group and 9 calves in the control group (2 bulls).

### Samples and laboratory examination

Serum was centrifuged for 15 min at 1900 x g and frozen at -80 °C for further analysis. In the serum samples, the concentration of immunoglobulins was determined in the IgM, IgG_1_, and IgG_2_ classes by ELISA (E10–101, E10–116, E10–117 Bethyl Laboratories, Montgomery, TX). For IgG_1_ determination, serum samples were diluted 80 000, 50 000, and 60 000x for S1, S2, and S3, respectively; for IgG_2,_ they were diluted 10 000x for S1, S2, and 30 000x for S3; for IgM, all examined samples were diluted 5000x. The anti-bovine immunoglobulin antibody conjugated with HRP (horseradish peroxidase) was diluted 60 000, 20 000, and 100 000x for IgG_1_, IgG_2,_ and IgM, respectively. The intra-assay CVs for IgG_1_, IgG_2_, and IgM were 3.6%, 1.9%, and 2.7%, respectively. The inter-assay CVs for IgG_1_, IgG_2_, and IgM were 18.9%, 24.3%, and 12.9%, respectively.

To evaluate the humoral immune response (in the IgG_1_, IgG_2_, and IgM classes) against vaccine antigens during cow hyperimmunization (recombinant *H. somni* Hsp60 and OMP40), an ELISA test was developed. Apart from analysing individual samples, the final pooled serum from the last blood collection after immunization was analyzed. ELISA was performed as previously described [13, 20] with some modifications.

Recombinant *H. somni* rHsp60 and rOMP40 proteins were produced by Pure Biologics S.A. [Wrocław, Poland]. Microplates (Nunc Maxisorp, F, USA) were coated with 100 µL per well for 2 h at 37 °C and at 4 °C overnight with *H. somni* rHsp60 (3 µg/mL in 0.05 M carbonate buffer pH = 9.6) or *H. somni* rOMP40 (3 µg/mL in phosphate-buffered saline PBS, pH = 7.4). Then, the wells were washed three times with 200 µl of PBS buffer containing 0.05% Tween 20 (Sigma‒Aldrich, USA, TPBS). Plates were blocked 200 µL/well for 75 min at 37 °C with 1% Tween 20 in PBS buffer. After blocking, the plates were washed 3 times with 200 µL of TPBS. For evaluation of the antibody reactivity with *H. somni* rHsp60, cow sera were diluted 10 000, 1000, and 500x (for IgG_1_, IgG_2_, and IgM class, respectively), and calf sera were diluted 1000x (for IgG_1_) and 100x (for IgG_2_ and IgM). For evaluation of the antibody reactivity with *H. somni* rOMP40, cow sera were diluted 4000, 1000, and 200x (IgG_1_, IgG_2_, IgM, respectively), and calf sera were diluted 100x for all examined classes. Then, the diluted serum was added (100 µL/well in duplicate) and incubated for 90 min at room temperature [22 ± 2^o^C]. After incubation, the plates were washed 3 times with 200 µL of TPBS. The secondary antibody was incubated for 60 min at room temperature (100 µL/well). For the IgG_1_ subclass, sheep anti-bovine IgG_1_ HRP conjugated (Bethyl Laboratories Inc., USA) diluted 60 000x was used. For IgG_2_ subclass sheep anti-bovine, IgG_2_ HRP conjugated (Bethyl laboratories Inc., USA) diluted 20 000x. For the IgM class, rabbit anti-bovine IgM HRP conjugated (Bethyl Laboratories Inc., USA) was diluted 100 000x. The reactions were developed with TMB Supersensitive Substrate (100 µL/well; Sigma‒Aldrich, USA) in the dark at room temperature for 20 min. The reaction was stopped by 2 M H_2_SO4 (100 µL/well, P.P.H. Stanlab Sp.J., Poland). Optical density was measured at a wavelength of 450 nm with an ELISA-Microplate Reader µQuantum™ (BioTek Instruments). To evaluate anti-rHsp60 and anti-rOMP40 reactivity, calf serum samples from S1 and S2 were analyzed on the same plate next to each other, in order as calves entered the trial. For S3 reactivity evaluation, samples from Limousine and MIX herds were evaluated on the same plate, and samples from Charolaise were evaluated on the other. The intra- and inter-assays for the reactivity of sera with recombinant proteins were calculated for absorbance.

The intra-assay CV for the reactivity of calf sera in IgG_1_, IgG_2_, and IgM with *H. somni* rHsp60 were 2.1%, 2.3%, and 1.8%, respectively. The intra-assay CV for the reactivity of IgG_1_, IgG_2_, and IgM with *H. somni* rOMP40 were 2.7%, 4.3%, and 2.7%, respectively. The inter-assay CV for reactivity (calculated for one serum sample evaluated on each plate) with *H. somni* rHsp60 in the IgG_1_, IgG_2_, and IgM classes were 21.8%, 21.6 and 19.1%, respectively. The inter-assay CV for reactivity with *H. somni* rOMP40 in the IgG_1_, IgG_2_, and IgM classes were 7.1%, 31.7%, and 24.5%, respectively.

Fibrinogen (Fb) was determined in whole blood according to Millar et al. [36]. The evaluation of serum concentration of acute phase proteins - haptoglobin (Hp, #2410–7 Life Diagnostics, Inc., Knypersley, UK) and serum amyloid A (SAA, TP 802, Tridelta Development, Maynooth, Ireland) was performed according to the manufacturer’s instructions. The inter-assay CV for SAA was 8.5%. The intra-assay CV for SAA was 6.6%. The inter-assay CV for Hp was 17.9%. The intra-assay CV for Hp was 15.4%.

The purity of the recombinant proteins used for immunization was evaluated by western blotting. The electrophoretic separation of the *H. somni* rHsp60 and rOMP40 protein preparations was performed in the SDS‒PAGE system under reducing conditions. After adding the dissociation buffer (4 x Laemmli Sample Buffer, Bio-Rad with 5% 2-mercaptoethanol), the samples were heated at 100 °C for 7 min and then centrifuged for 10 min at 10 000xg. Samples were separated by SDS‒PAGE in a 10% polyacrylamide gel (20 mA per gel; ~1.5 µg proteins per lane). Then, the proteins were transferred onto 0.45 μm nitrocellulose membranes (Bio-Rad) using a Trans-Blot Turbo Biorad semidry chamber (transfer conditions: 25 V, 1.0 A, 30 min). Later, the membrane was blocked using SEA Block Blocking Buffer (Thermo Scientific; blocking conditions: 150 min at 37 °C). After blocking, the membrane was incubated with the primary antibody mouse anti-HisTag monoclonal antibody (Clontech) diluted 2 000x in TBS [pH = 8.3] with 0.05% Tween 20 (TBST) overnight at 4 °C. Then, the membrane was washed (three times for 5 min) and incubated with secondary antibodies: rabbit anti-mouse IgG HRP conjugated (Sigma‒Aldrich, USA) diluted 160 000x in TBST for 90 at room temperature. The reaction was developed with Clarity Western ECL (Bio-Rad) as the substrate. Membrane visualization was performed using ChemiDoc Touch Instruments. Molecular weights of proteins were estimated using Image Lab™ software by comparison with a standard protein marker (Spectra™ Multicolor Broad Range Protein Ladder, Fermentas).

### Statistical analysis

Statistical analyses were carried out on Microsoft Excel spreadsheets and STATISTICA software v.12.5 (StatSoft Inc., Tulsa, OK, USA). Data were evaluated for normality with the Liliefors test. In cases of abnormal distribution, logarithmic transformations were used for the evaluation of normality. In cases where normality was not improved, nonparametric tests were used. As the initial antibody reactivity of cows was similar in different Ig classes, for statistical analysis, all immunized cows from different herds were merged into one group. For analysis, absorbance results from double repetitions were studied. Therefore, in each sampling, eight results from four cows were in the group. Data from the immunization of cows were compared with Friedman ANOVA with the Wilcoxon Matched Paired test, and the first sampling was compared to subsequent samplings.

In calves, the results of antibody reactivity against *H. somni* rHsp60 and rOMP40 in the IgG_1_, IgG_1_, and IgM classes, the concentration of immunoglobulins in the G_1_ and M classes, haptoglobin, serum amyloid A, rectal temperature, number of respiratory rates, and heart rate were compared between groups separately for each herd and sampling with the Mann‒Whitney U test.

Fibrinogen concentration and IgG_2_ concentration (log10 results) were analyzed in a split plot ANOVA design (herd, group as independent variable, and sampling as repeated measures).

The average daily gain in the first month and above the first month of weaning was compared in factorial ANOVA (herd, group, and calf sex).

### Electronic supplementary material

Below is the link to the electronic supplementary material.

Additional file 1: The raw image of Fig. 1

## Data Availability

All data generated or analyzed during this study are included in this article and are available from the corresponding author upon reasonable request.

## Abbreviations

HS: Bovine hyperimmune serum
CH: Charolaise
LM: Limousine
MIX: Crossbreed beef and dairy breed
C: Control
E: Experimental
ADG: Average daily gain
