# Correction: Subcutaneous application of hyperimmune serum against *Histophilus somni* recombinant proteins affects serum antibody reactivity in beef calves

**DOI:** 10.1186/s12917-024-04038-3

**Published:** 2024-05-07

**Authors:** Joanna Bajzert, Paulina Jawor, Rafał Baran, Tadeusz Stefaniak

**Affiliations:** Department of Immunology, Pathophysiology and Veterinary Preventive Medicine, Wroclaw University of Environmental and Life Sciences, C.K. Norwida 31 Str, Wrocław, 50-375 Poland

**Correction**: ***BMC Vet Res*20, 51 (2024)**

10.1186/s12917-024-03895-2

Following publication of the original article [1], the authors identified an error in the author list. The given names and family names were erroneously transposed. The original article has been corrected.

The incorrect author list:

Bajzert Joanna^1^, Jawor Paulina^1*^, Baran Rafał^1^ and Stefaniak Tadeusz^1^.

The correct author list:

Joanna Bajzert^1^, Paulina Jawor^1*^, Rafał Baran^1^ and Tadeusz Stefaniak^1^.
