# Supplementary figures and images for: Subcutaneous application of hyperimmune serum against Histophilus somni recombinant proteins affects serum antibody reactivity in beef calves
Source: BMC Vet Res. 2024 Feb 10;20:51. doi: 10.1186/s12917-024-03895-2 (PMC10858532; doi:10.1186/s12917-024-03895-2)

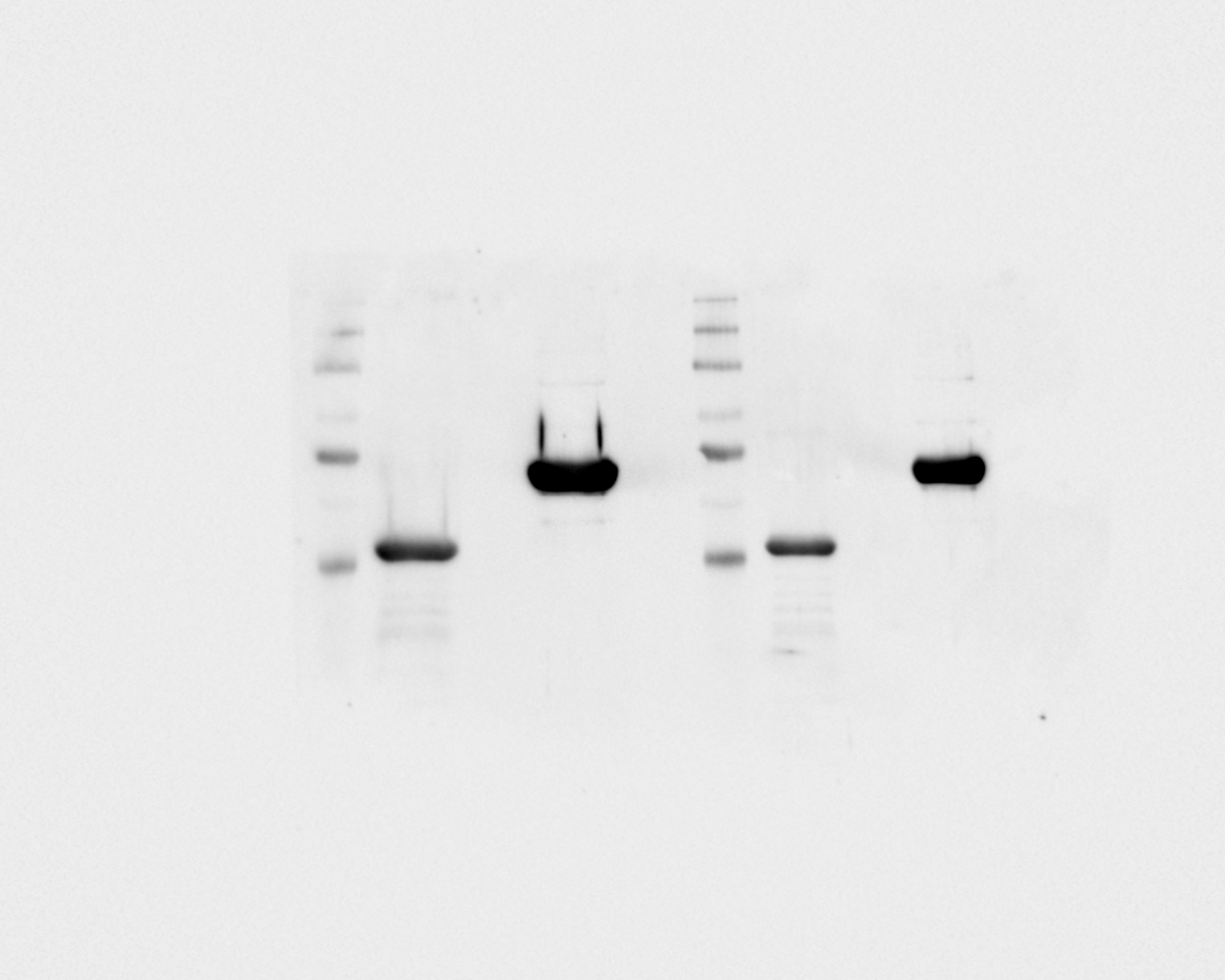

Supplement: Supplementary file 1 — Additional file 1: The raw image of Fig. 1 [file 12917_2024_3895_MOESM1_ESM.jpg]
